# Quantitative Analysis of Bioactive Phenanthrenes in *Dioscorea batatas* Decne Peel, a Discarded Biomass from Postharvest Processing

**DOI:** 10.3390/antiox8110541

**Published:** 2019-11-10

**Authors:** Minyoul Kim, Myeong Ju Gu, Joon-Goo Lee, Jungwook Chin, Jong-Sup Bae, Dongyup Hahn

**Affiliations:** 1 School of Food Science and Biotechnology, College of Agriculture and Life Sciences, Kyungpook National University, Daegu 41566, Korea; 2 Department of Integrative Biology, Kyungpook National University, Daegu 41566, Korea; 3 Food Standard Division, Ministry of Food and Drug Safety, Cheongju 28159, Korea; 4 New Drug Development Center, Daegu-Gyeongbuk Medical Innovation Foundation, Daegu 41061, Korea; 5 College of Pharmacy, CMRI, Research Institute of Pharmaceutical Sciences, BK21 Plus KNU Multi-Omics based Creative Drug Research Team, Kyungpook National University, Daegu 41566, Korea; 6 Institute of Agricultural Science and Technology, College of Agriculture and Life Sciences, Kyungpook National University, Daegu 41566, Korea

**Keywords:** *Dioscorea batatas*, Chinese yam, phenanthrenes, quantitative analysis

## Abstract

*Dioscorea batatas* Decne (Chinese yam) has been widely cultivated in East Asia for the purposes of food and medicinal uses for centuries. Along with its high nutritional value, the medicinal value of *D. batatas* has been extensively investigated in association with phytochemicals such as allantoin, flavonoids, saponins and phenanthrenes. Phenanthrenes are especially considered the standard marker chemicals of the Chinese yam for their potent bioactivity and availability of analysis with conventional high performance liquid chromatography with ultraviolet detection (HPLC-UV) methods. In order to investigate how much the contents of phenanthrenes are in the actual food products provided for consumers, *D. batatas* tuber was peeled and separated into its peel and flesh as in the conventional processing method. A quantitative analysis using the HPLC-UV method revealed that phenanthrenes are concentrically present in the *D. batatas* peel, while phenanthrenes are present in the flesh under the limit of detection. The difference in the contents of phenanthrenes is estimated to have arisen the considerable difference of antioxidant potential between the peel and the flesh. The results from this study suggest the high value of the discarded biomass of the Chinese yam peel and the necessity for the utilization of the Chinese yam peel.

## 1. Introduction

*Dioscorea batatas* Decne (Chinese yam) is a perennial plant widely cultivated across the tropical and subtropical regions of East Asia as a staple or as a traditional medicine [1]. It has high nutritional values because it contains substantial proteins, carbohydrates, vitamins, fats, choline, and indispensable trace elements for the human body such as iodine, iron, calcium and phosphorus [2]. In addition, its medicinal value is significant because it contains numerous bioactive constituents such as polysaccharides, allantoin, and polyphenols including flavonoids [3]. Previous studies have demonstrated that the Chinese yam exerts strong anti-oxidative [4], cholesterol-lowering [5], growth hormone-releasing activity [6], and protective effects against ethanol-induced gastric ulcers [7]. Recent studies have revealed intimate relations between bioactivities and their responsible secondary metabolites including allantoin, saponins and phenanthrenes. Allantoin has been reported to promote wound healing, the speeding up of cell regeneration, and the exhibition of a keratolytic effect [8]. Saponins from the Chinese yam are reported to have anticancer and fungistatic activity [9]. Phenanthrenes are a representative class of phenolic phytochemicals found in the Dioscoreaceae family. They are known to be biosynthesized from the oxidative coupling of the aromatic rings of stilbene precursors [10] by the tubers, roots, and stems of only 17 taxonomical families of plants [11]. Phenanthrene-containing plants including the *Dioscorea* genus have been widely used for the treatment of several diseases in Africa, Asia, and South America [11]. Since the discovery of antifungal phenanthrenes from *D. rotundata* [12], anti-inflammatory [6,13,14], anticholinesterase [15], and triglyceride accumulation inhibitory [16] activity have been reported as medicinal or health-promoting effects of phenanthrenes discovered from the *Dioscorea* genus. In addition to their bioactivity, phenanthrenes have been suggested to be a non-polar standard marker for the *Dioscorea* genus, as their structures are distinguished from common phytochemicals and they are not difficult to analyze with the conventional high performance liquid chromatography with ultraviolet detection (HPLC-UV) method compared to other phytochemicals found in the *Dioscorea* genus such as allantoin and saponins [17].

Our previous studies on biological activities of *D. batatas* also revealed anti-inflammatory [18] and inhibitory effects on the particulate matter-induced pulmonary injury of phenanthrenes [19]. On the basis of cumulative research, phenanthrenes are putative medicinal components in *D. batatas*. However, how much the contents of phenanthrenes are in actual food products provided for consumers of *D. batatas* has been overlooked. The most common postharvest processing of *D. batatas* tuber is peeling, drying under heat, and then pulverizing the dried flesh to make ‘yam flour,’ which yields discarded peels without utilization [20]; or *D. batatas* is deteriorated so quickly that only a small fraction of harvested *D. batatas* is supplied as raw products, and most of the Chinese yam is consumed as ‘yam flour’ or products from the flour [21]. The aim of this study was to verify if phenanthrenes, the polyphenolic bioactive components, are properly provided to consumers of the Chinese yam by investigating the contents of three representative phenanthrenes in the *D. batatas* flesh (DBF), an edible portion, and the *D. batatas* peel (DBP), a discarded byproduct.

## 2. Materials and Methods

### 2.1. Plant Material

The Chinese yam (*D. batatas*) was purchased from Taesan-nongjang (Andong, Korea). The Chinese yam tubers were peeled off, and then the flesh and the peel were separated. The peel was washed with water. Then, both the flesh and the peel were cut into slices and dried with a freeze-dryer (Ilshinbiobase, Dongducheon, Korea).

### 2.2. Chemicals and Reagents

Ethyl acetate (EA, extra pure grade), dichloromethane (DCM, extra pure grade), methanol (MeOH, extra pure grade), hexanes (extra pure grade) and butanol (extra pure grade) were purchased from Duksan pure chemicals Co. (Ansan, Korea). Acetonitrile (reagent grade), water (reagent grade) and methanol (reagent grade) were purchased from J.T.Baker (Phillipsburg, NJ, USA). Acetone-d6 (deuteration degree min. 99.9%) and dimethyl sulfoxide- *d*_6_ (deuteration degree min. 99.8%) for NMR spectroscopy were purchased from Merck (Darmstadt, Germany). Trifluoroacetic acid (TFA), 1,1-diphenyl-2-picryl hydrazyl (DPPH), 2,2-azino-bis-(3-ethylbenzothiazoline-6-sulfonic acid), and diammonium salt (ABTS+) were purchased from Sigma-Aldrich (St Louis, MO, USA).

### 2.3. Preparation of Standard Phenanthrene Compounds

**Figure 1:**
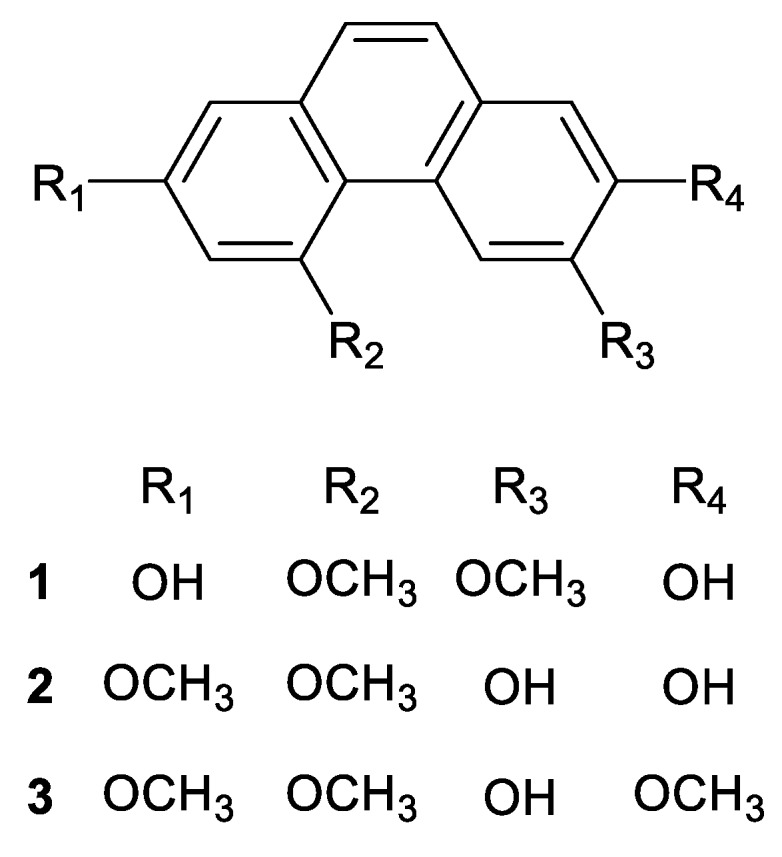
Structures of Phenanthrenes **1**–**3.**

Phenanthrene Compounds **1**, **2** and **3** (Figure 1) were isolated from the peel of *D. batatas* following the method suggested by our previous studies [18,19]. The peel was extracted with 95% ethanol for 48 h, and solvents were removed in vacuo. The ethanol extract was partitioned into n-hexane, ethyl acetate, butanol and water layers. The ethyl acetate layer was dissolved in mixture of DCM:MeOH (1:1, *v*/*v*) and fractioned on a size exclusion chromatography column filled with Sephadex^®^ LH20 resin (Pharmacia, Stockholm, Sweden) using DCM:MeOH (1:1, *v*/*v*) as a mobile phase. Among the thirteen fractions yielded, the eighth fraction was further separated with a high performance liquid chromatograph (Waters 1525 system, Waters, Milford, MA, USA) equipped with a dual absorbance ultraviolet detector (Waters 2487, Waters). A gradient mixture of acetonitrile/water (39:61 (0–44 min) → 100:0 (44.01–60 min)) was used as a mobile phase, and a Hector-A-C18 column (250 × 10.0 mm, 5 μm, RStech corporation, Daejeon, Korea) was used to isolate Compounds **1** (2,7-dihydroxy-4,6-dimethoxy phenanthrene), **2** (6,7-dihydroxy-2,4-dimethoxy phenanthrene). Compound **3** was isolated from the ninth fraction from LH20 column chromatography by using the same HPLC condition for the isolation of Compounds **1** and **2**. The structures of Compounds **1** and **2** were identified by our previous study [18,19] on the basis of ^1^H- and ^13^C-NMR spectra (Figures S1 and S2), and the structure of Compound **3** as identified as 6-hydroxy-2,4,7-trimethoxyphenanthrene (batatasin I) through a comparison of ^1^H and ^13^C NMR spectra (Figure S3) with a previous report [22]. NMR experiments were carried out using a Bruker Ascend (^1^H-500 MHz, ^13^C-125 MHz, Billerica, MA, USA) spectrometer. 

### 2.4. Samples and Standard Solutions Preparation

The freeze-dried *D. batatas* flesh (DBF) and *D. batatas* peel (DBP) were powdered, and 1 g of dried powder from each sample was extracted at 25 °C with 250 mL of 95% ethanol for 12 h, followed by filtration with 0.45 μm syringe filter (Advantec, Tokyo, Japan) and evaporation in vacuo. The resultant extracts were weighed and dissolved in 10 mL of methanol. The stock solutions of Compounds **1**–**3** were prepared by dissolving Compounds **1**–**3** in methanol to yield 1 mg/mL. Standard solutions were prepared by the serial dilution of the stock solutions to methanol. The range of the standard solutions was 3.125–100 µg/mL (3.125, 6.25, 12.5, 25, 50 and 100 µg/mL). All of the standard solutions were stored at −20 °C in darkness until analysis. 

### 2.5. Quantitative Analysis of Phenanthrenes with HPLC

The quantitative analysis of Phenanthrene Compounds **1**–**3** was performed using an Alliance 2695 HPLC system (Waters) with a photodiode array detector (Waters 2996, Waters) and a Hector-M-C18 column (250 × 4.6 mm, 5 μm, RStech). The overall method was modified from the method using HPLC-UV suggested by Yoon et al. [17]. The mobile phases used were 0.1% trifluoroacetic acid in water (A) and acetonitrile (B) at a flow rate of 1.0 mL/min. The gradient program was set as follows: 0–5 min, 5% B; 5–20 min, 5%–100% B; and 20–25 min, 100% B. The column temperature was maintained at 40 °C throughout the analysis. The chromatograms were monitored at a wavelength of 260 nm, and the injection volume was 10 μL. The DBF and DBP samples were injected 3 times each, and the averages of the peak areas on the chromatograms were obtained for quantitative analysis. Calibration curves of each compounds were obtained by averaging the peak areas of each compound on the chromatograms acquired from 3 injections that were monitored at a wavelength of 260 nm. The limit of detection (LOD) and the limit of quantification (LOQ) under the chromatographic conditions were determined by the serial dilution of the standard solution on the basis of a signal to noise (S/N) ratio of 3 to 10.

### 2.6. Determination of DPPH Radical Scavenging Activity

The DPPH radical scavenging activity of the extracts and compounds were measured as described by Kirigaya et al. [23] with slight modifications. Twenty microliters of each extract were mixed with 80 µL of the 0.2 mM DPPH radical methanolic solution. After incubation at 37 °C for 30 min in darkness, the absorbance of the mixture was measured at 515 nm using a Sunrise^TM^ microplate reader (Tecan Group Ltd., Männedorf, Switzerland). To evaluate the radical scavenging activity of Compounds **1**–**3**, each compound was dissolved in 70%ethanol (*v*/*v*) at concentrations of 0.0125–1.000 mg/mL. For extracts, DBP and DBF, they were dissolved in 70% ethanol (*v*/*v*) at concentrations of 0.125–10.000 mg/mL. *L*-ascorbic acid in 70% ethanol (*v*/*v*) was used as a positive control at concentrations of 0.0125–0.1000 mg/mL. The effective concentrations of the compounds or extracts required to scavenge DPPH radical by 50% (IC_50_) were obtained by a linear regression analysis of the dose–response curve plotting between %inhibition and concentrations. The DPPH radical scavenging activity at every concentration of each compound or extract was documented in Table S1.

### 2.7. ABTS+ Radical Scavenging Activity

The radical cations were prepared by mixing a 7 mM ABTS+ stock solution in water with 2.45 mM potassium persulfate and stored in the dark for 12–16 h at room temperature. The ABTS+ solution was diluted with absolute ethanol to an absorbance of 0.7 ± 0.02 at 734 nm before use. In a 96-well plate, 20 µL of each extract was mixed with an 80 µL ABTS+ solution. Measurements were taken at 734 nm using a Sunrise^TM^ microplate reader (Tecan Group Ltd.). To evaluate the radical scavenging activity of Compounds **1**–**3**, each compound was dissolved in 70% ethanol (*v*/*v*) at concentrations of 0.0125–1.000 mg/mL. *L*-ascorbic acid in 70% ethanol (*v*/*v*) was used as a positive control at concentrations of 0.0125–0.1000 mg/mL. The ABTS+ radical scavenging activity of the DBP and DBF extracts dissolved in 70% ethanol (*v*/*v*) at concentrations of 0.125–10.000 mg/mL. The effective concentrations of the compounds or extracts required to scavenge ABTS+ radical by 50% (IC_50_) were obtained by a linear regression analysis of the dose–response curve plotting between %inhibition and concentrations. The ABTS+ radical scavenging activity at every concentration of each compound or extract was documented in Table S2.

### 2.8. Statistical Analysis 

The experimental results are presented as mean and standard deviation (mean ± SD). All the experiments were analyzed in triplicated measurements. The one-way analysis of variance (ANOVA) was run using the SPSS ver. 23.0 software (SPSS Inc., Chicago, IL, USA), and the Duncan’s multiple range test comparisons at *p* < 0.05 were run to determine significant difference.

## 3. Results and Discussion

### 3.1. Calibration, Limit of Detection (LOD) and Limit of Quantification (LOQ)

The method used in this study was an HPLC-UV method that monitored phenanthrene derivatives at the wavelength of 260 nm. A similar method was suggested and validated by Yun et al. [17]. In this study, linearity, the LOD and the LOQ were validated. For the preparation of the calibration curve, standard solutions were prepared by serial dilution to appropriate concentrations. The linearity of calibration curves obtained from standard solutions was satisfactory with the determination coefficients (R^2^), which were greater than 0.9959 in the concentration range of 3.125–100 μg/mL (Table 1). The LODs of standard phenanthrenes were smaller than 0.58 μg/mL, and the LOQs were smaller than 1.94 μg/mL (Table 1). 

### 3.2. Quantitative Analysis of Phenanthrenes with HPLC

**Figure 2:**
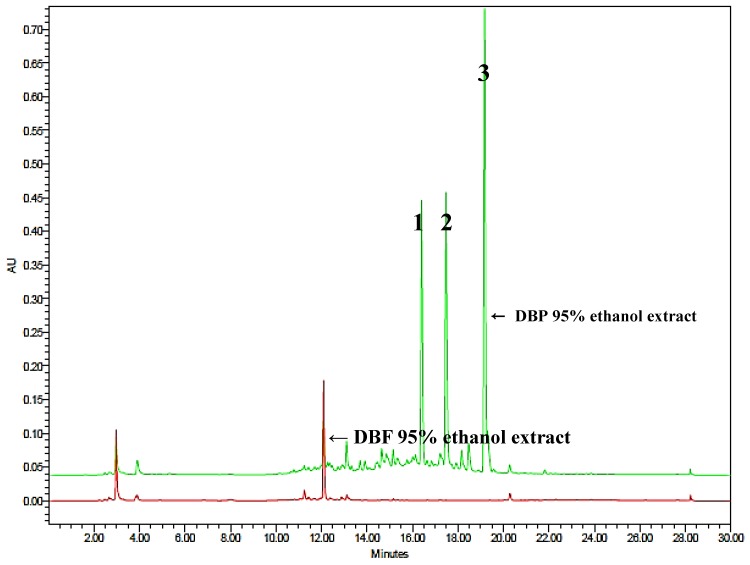
HPLC chromatogram of the *Dioscorea batatas* flesh (DBF) (red) and the *D. batatas* peel (DBP) (green) extracts monitored at the wavelength of 260 nm.

Chemical screening for phenanthrenes present in the DBP and DBF extracts by monitoring the HPLC chromatograms of 260 nm wavelength detection revealed that Compounds **1**–**3** are major phenanthrenes present in the DBP, but the DBF scarcely contained any type of phenanthrenes (Figure 2). The chromatogram of the DBF extract exhibited a peak at the retention time (r.t.) of 12.10 min, and its UV absorption spectrum was completely different from those of phenanthrenes (data not shown). According to the chromatograms, Compounds **1**–**3** were completely separated without overlapping with other peaks. The retention times (r.t.) of Compounds **1**–**3** were 16.4, 17.5 and 19.2 min, respectively. 

By the subsequent quantitative analysis with HPLC, the content of Compound **1** in the DBP was 47.35 ± 0.25 mg/100 g on the dry weight basis and 6.76 ± 0.04 mg/100 g on the wet weight basis; the content of Compound **2** was 29.29 ± 0.08 mg/100 g on the dry wt. basis and 4.18 ± 0.01 mg/100 g on the wet wt. basis; the content of Compound **3** was 35.85 ± 0.12 mg/100 g on the dry wt. basis and 5.12 ± 0.02 mg/100 g on the wet wt. basis. However, it was not possible to assess the contents of any phenanthrenes in the DBF extract, as the DBF contained only trace amounts under the detection limit values of Phenanthrenes **1**–**3** (Table 2). There already have been attempts to investigate differences in antioxidant activity and total phenolic contents between the flesh and the peel of the Chinese yam [24,25]. Both of these research articles reported that the Chinese yam peel exhibited more potent antioxidant activity and more total phenolic contents than the flesh. The study of Liu et al. [25] also revealed a significant difference between total phenolics and total flavonoids contents in the peel, which implies that the peel contains even more amounts of non-flavonoidal phenolics than flavonoids, at least by five folds. Their study did not identify the responsible non-flavonoidal phenolic compounds that account for the difference. However, from our analytical result, it is now comprehensible to presume that phenanthrenes may largely contribute to the phenolics contents in the Chinese yam peel.

### 3.3. Antioxidant Activity of Phenanthrenes, DBP and DBF

**Figure 3:**
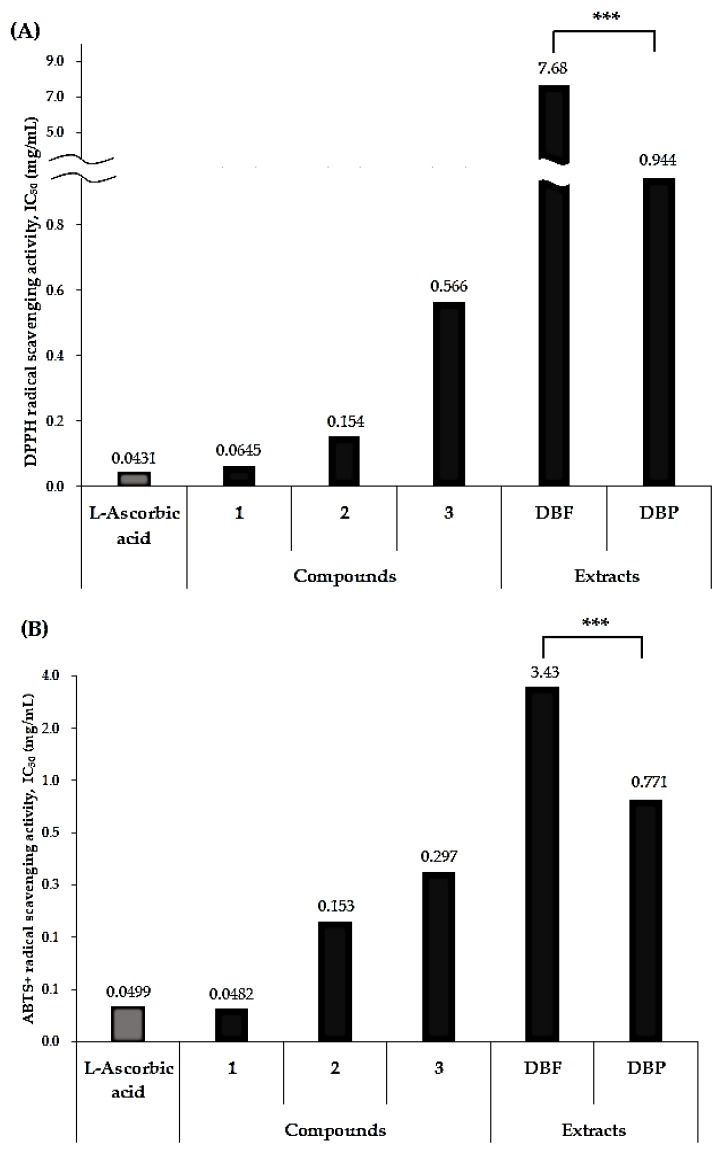
Antioxidant activities of Compounds **1**–**3** for the DBP and DBF extracts. The DPPH (diphenyl-2-picryl hydrazyl) (**A**) and ABTS+ (diammonium salt) (**B**) radical scavenging activities for Compounds **1**–**3**, the DBP, the DBF extracts, and the positive control (l-ascorbic acid) were measured in IC_50_ (mg/mL). *** *p* < 0.001.

As presented in Figure 3, Compound **1** exhibited the strongest antioxidant activity among the phenanthrenes isolated from *D. batatas*. Compound **1** exhibited strong DPPH radical scavenging activity with an IC_50_ value of 0.0645 mg/mL and an even stronger ABTS+ inhibition with an IC_50_ value of 0.0482 mg/mL, which was equivalent to the ABTS+ inhibition of the positive control, *L*-ascorbic acid (IC_50_ 0.499 mg/mL). Compounds **2** and **3** also exhibited relatively weaker antioxidant activity compared to Compound **1** and *L*-ascorbic acid. Compound **2** exhibited DPPH and ABTS+ radical scavenging activity with IC_50_ values of 0.154 and 0.153 mg/mL, respectively. Compound **3** exhibited DPPH and ABTS+ radical scavenging activity with IC_50_ values of 0.566 and 0.297 mg/mL, respectively. The strong antioxidant activity of Compound **1** is not surprising, as it has been reported to be an antioxidant [14], an antifungal [26] and a pancreatic lipase inhibitory agent [27] isolated from plants that belong to *Dioscorea* sp. cultivated in Korea. Our previous study on the anti-inflammatory activity of *D. batatas* [18] also led to the isolation of Compound **1** as a responsible component for the bioactivity.

In accordance with previous studies that have revealed the difference of antioxidant activity between the peel and the flesh of the Chinese yam [24,25], the DPPH and ABTS+ scavenging assay exhibited far more potent antioxidant activity of the DBP compared to that of the DBF (Figure 3). Both the DPPH and ABTS+ radical scavenging assays showed a wide gap in antioxidant activity between the DBP and the DBF (Figure 3). The DBP extract dissolved in 70% ethanol exhibited inhibition against DPPH and ABTS+ with IC_50_ values of 0.944 and 0.771 mg/mL, respectively. On the other hand, the DBF exhibited far weaker antioxidant activity, showing inhibition against DPPH with an IC_50_ value of 7.68 mg/mL and inhibition against ABTS+ with an IC_50_ value of 3.43 mg/mL. A previous report by Liu et al. [25] doubtfully concluded that the more potent antioxidant activity of the peel might be attributed to the slightly higher content of allantoin in the peel than in the flesh. However, the results from this study imply that the difference in the content of phenanthrenes, antioxidant agents as potent as the positive control *L*-ascorbic acid, is the crucial factor for the large difference in the antioxidant activity between the DBP and the DBF. 

## 4. Conclusions

In practice, the Chinese yam peel has been regarded as inedible due to its soil contents, and it has been discarded without utilization. As the Chinese yam has a low preservation potential, most of its harvest biomass is to be peeled, dried, and powdered. The analysis of phenanthrene contents in the DBP compared to the DBF reflects that consumers hardly ingest potent antioxidant agents suchas phenanthrenes by consuming products from the Chinese yam flour made by conventional postharvest processing. However, the Chinese yam peel has now been proven a valuable resource for bioactive components with high potentials for pharmaceuticals or functional biomaterials. In conclusion, a quantitative analysis of phenanthrenes, representative phenolic natural products with high antioxidant and functional potentials, was performed using the HPLC-UV method, which revealed that the contents in the DBP are significantly higher than in the DBF. The difference in the contents of phenanthrenes is estimated to result in the considerable difference of antioxidant potential between the DBP and the DBF. Further to the results from this study, it is strongly urged to investigate the appropriate means to optimize the utilization of the discarded biomass of the Chinese yam peel.

## Figures and Tables

**Table 1:** Calibration parameters for the HPLC method.

Compound	Regression Equation	Range (µg/mL)	*R^2^*	LOD (µg/mL)	LOQ (µg/mL)
**1**	Y = 301.34x − 46,588	3.125–100	0.9994	0.44	1.47
**2**	Y = 510.71x − 21,040	3.125–100	0.9990	0.58	1.94
**3**	Y = 99.155x + 15,3981	3.125–100	0.9959	0.42	1.40

**Table 2:** Contents of Phenanthrenes **1**–**3** in the DBF and DBP 95% ethanol extracts.

		Content (mg/100 g)			
		DBP		DBF	
		Dry wt. Basis	Wet wt. Basis	Dry wt. Basis	Wet wt. Basis
**Compound**	**1**	47.35 ± 0.25	6.76 ± 0.04	N.D.	N.D.
	**2**	29.29 ± 0.08	4.18 ± 0.01	N.D.	N.D.
	**3**	35.85 ± 0.12	5.12 ± 0.02	N.D.	N.D.

* Values represent the means ± SD (*n* = 3).

## Supplementary Materials

The following are available online at https://www.mdpi.com/2076-3921/8/11/541/s1, Figure S1: ^1^H and ^13^C NMR spectrum of Compound **1**, Figure S2: ^1^H and ^13^C NMR spectrum of Compound **2**, Figure S3: ^1^H and ^13^C NMR spectrum of Compound **3**, Table S1: DPPH radical scavenging activity of Compounds **1–3**, the DBP and DBF extracts., Table S2: ABTS+ radical scavenging activity of Compounds **1–3**, the DBP and DBF extracts.
