# Supplementary material for: Quantitative Analysis of Bioactive Phenanthrenes in Dioscorea batatas Decne Peel, a Discarded Biomass from Postharvest Processing
Source: Antioxidants (Basel). 2019 Nov 10;8(11):541. doi: 10.3390/antiox8110541 (PMC6912652; doi:10.3390/antiox8110541)
Supplement: Supplementary file 1 [file antioxidants-08-00541-s001.pdf]

Supporting Information

Quantitative Analysis of Bioactive Phenanthrenes in *Dioscorea batatas* Decne Peel, a Discarded Biomass from Postharvest Processing

Minyoul Kim,[1,2,†] Myeong Ju Gu,[1,†] Joon-Goo Lee,[3] Jungwook Chin,[4] Jong-Sup Bae,[5,*] and Dongyup Hahn,[1,2,6,*]

[1] School of Food Science and Biotechnology, College of Agriculture and Life Sciences, Kyungpook National University, Daegu 41566, Republic of Korea
[2] Department of Integrative Biology, Kyungpook National University, Daegu 41566, Republic of Korea
[3] Food Standard Division, Ministry of Food and Drug Safety, Cheongju 28159, Republic of Korea
[4] New Drug Development Center, Daegu-Gyeongbuk Medical Innovation Foundation, Daegu 41061, Republic of Korea
[5] College of Pharmacy, CMRI, Research Institute of Pharmaceutical Sciences, BK21 Plus KNU Multi-Omics based Creative Drug Research Team, Kyungpook National University, Daegu 41566, Repulic of Korea
[6] Institute of Agricultural Science and Technology, College of Agriculture and Life Sciences, Kyungpook National University, Daegu 41566, Republic of Korea
* Correspondence: dohahn@knu.ac.kr (D.H.); baejs@knu.ac.kr (J.-S.B.)
†These authors contributed equally to this work

Contents

1) **Figure S1**. ^{1}H and ^{13}C NMR spectra of compound **1** (^{1}H-500MHz, ^{13}C-125MHz, Acetone-d_6)

2) **Figure S2**. ^{1}H and ^{13}C NMR spectra of compound **2** (^{1}H-500MHz, ^{13}C-125MHz, Acetone-d_6)

3) **Figure S3**. ^{1}H and ^{13}C NMR spectra of compound **3** (^{1}H-500MHz, ^{13}C-125MHz, Acetone-d_6)

4) **Table S1.** DPPH radical scavenging activity of compounds **1-3**, DBP and DBF extracts.

5) **Table S2.** ABTS+ radical scavenging activity of compounds **1-3**, DBP and DBF extracts.

Figure S1. ^{1}H and ^{13}C NMR spectra of compound 1 (^{1}H-500MHz, ^{13}C-125MHz, Acetone-d_6)

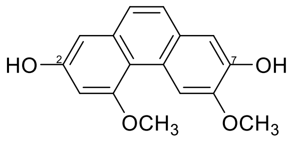

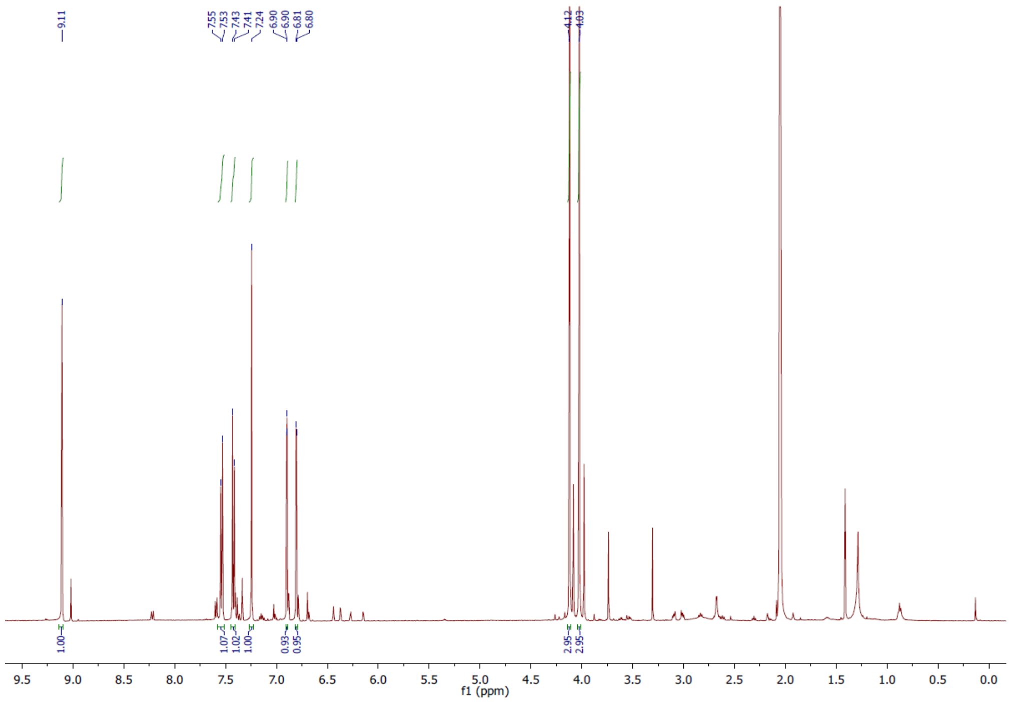

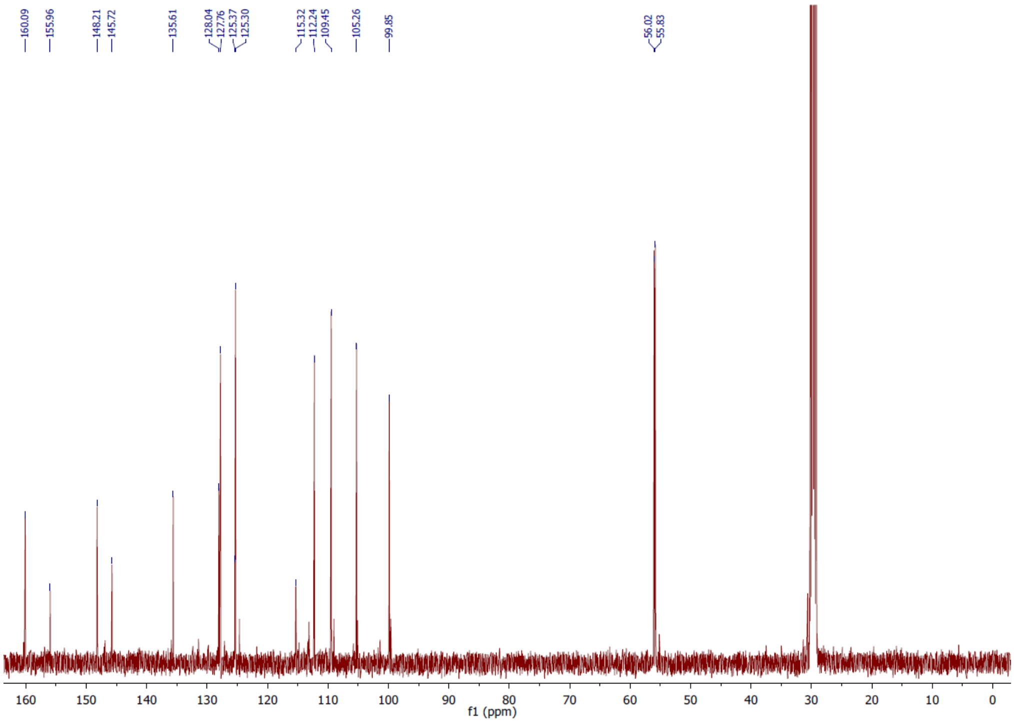

Figure S2. ^{1}H and ^{13}C NMR spectra of compound 2 (^{1}H-500MHz, ^{13}C-125MHz, Acetone-d_6)

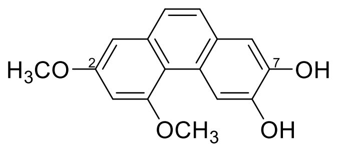

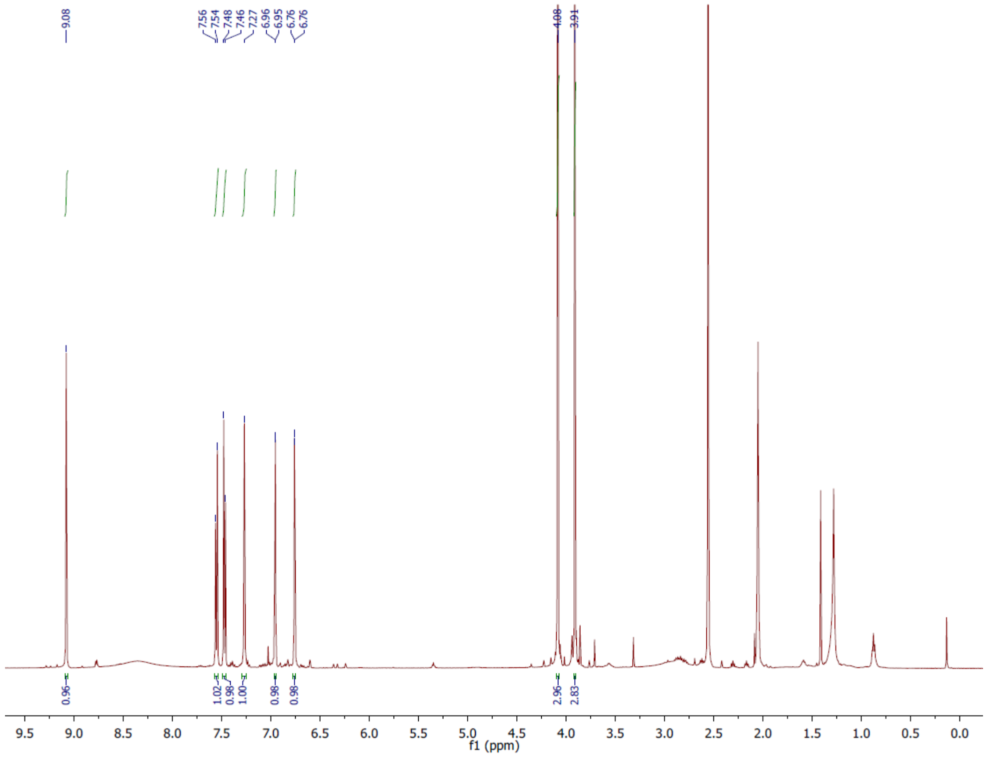

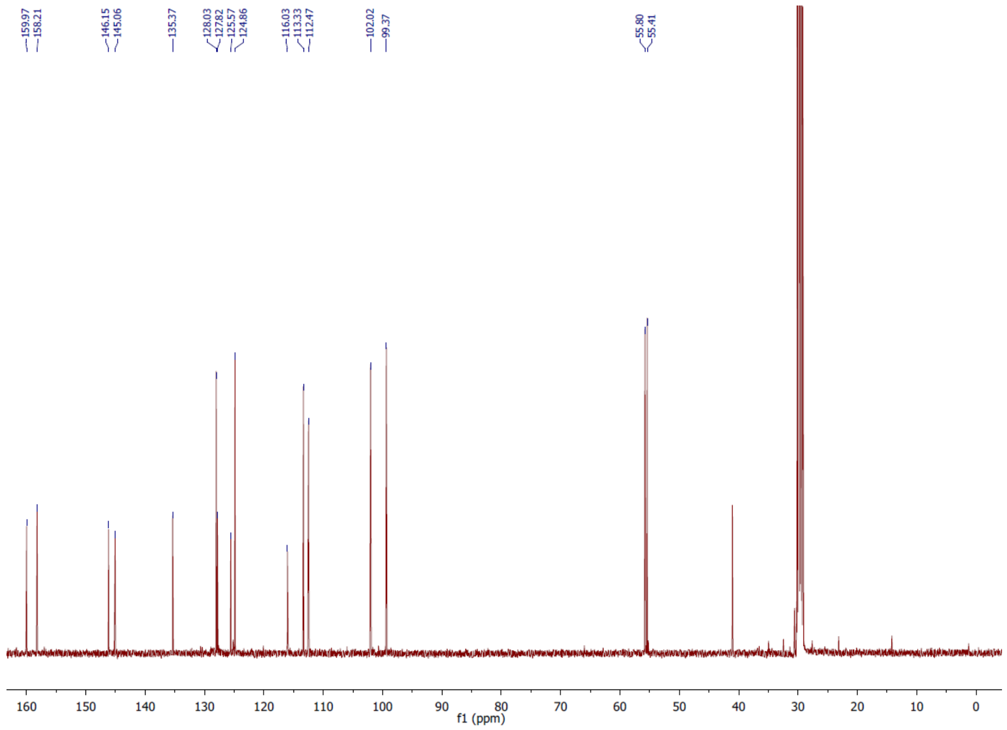

Figure S3. **^{1}H and ^{13}C NMR spectra of compound 3 (^{1}H-500MHz, ^{13}C-125MHz, DMSO-d_6)**

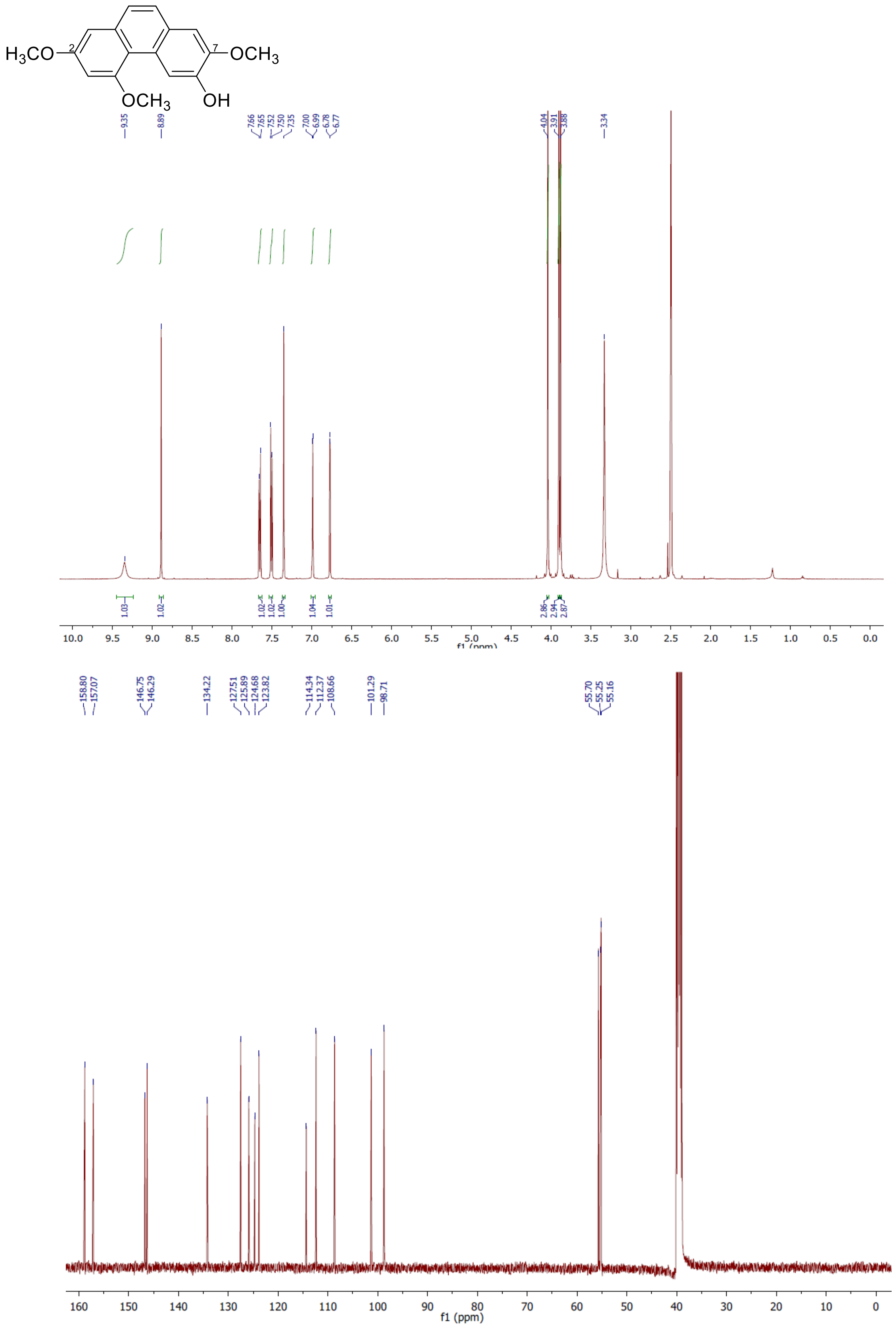

Table S1. DPPH radical scavenging activity of compounds **1-3**, DBP and DBF extracts.

Sample	Concentration (mg/mL)	% Reduction	IC_{50} (mg/mL)
L-Ascorbic acid	0.1000	91.35 ± 0.82	0.0431
	0.0500	56.33 ± 1.24	
	0.0250	41.70 ± 3.75	
	0.0125	21.83 ± 1.44	
**1**	0.1000	79.34 ± 6.28	0.0645
	0.0500	34.93 ± 0.27	
	0.0250	22.14 ± 1.02	
	0.0125	7.34 ± 3.63	
**2**	0.2500	83.54 ± 2.16	0.1543
	0.1250	40.16 ± 4.74	
	0.0625	17.64 ± 2.59	
	0.0313	6.42 ± 1.02	
**3**	1.0000	72.62 ± 6.02	0.5656
	0.5000	57.95 ± 0.69	
	0.2500	35.76 ± 1.02	
	0.1250	7.95 ± 0.99	
DBF	10.000	61.22 ± 2.53	7.6758
	5.000	40.96 ± 4.13	
	2.500	18.17 ± 4.32	
	1.250	3.23 ± 3.42	
DBP	1.000	52.31 ± 2.14	0.9441
	0.500	26.46 ± 6.33	
	0.250	9.08 ± 1.64	
	0.125	1.27 ± 1.25	

Table S2. ABTS+ radical scavenging activity of compounds **1-3**, DBP and DBF extracts.

Sample	Concentration (mg/mL)	% Reduction	IC_{50} (mg/mL)
L-Ascorbic acid	0.1000	91.80 ± 0.30	0.0499
	0.0500	58.58 ± 7.62	
	0.0250	32.39 ± 4.67	
	0.0125	6.24 ± 4.99	
**1**	0.1000	88.76 ± 1.04	0.0482
	0.0500	64.35 ± 1.62	
	0.0250	29.68 ± 3.17	
	0.0125	12.55 ± 0.64	
**2**	0.2000	56.76 ±6.16	0.1534
	0.1000	44.78 ± 3.72	
	0.0500	32.10 ± 2.79	
	0.0250	14.34 ± 0.68	
**3**	1.0000	86.68 ± 0.57	0.2965
	0.5000	79.98 ± 1.23	
	0.2500	40.83 ± 4.38	
	0.1250	34.87 ± 7.55	
DBF	10.000	92.27 ± 0.01	3.4269
	5.000	75.45 ± 1.44	
	2.500	43.82 ± 3.34	
	1.250	25.52 ± 2.16	
DBP	1.000	64.41 ± 2.57	0.7708
	0.500	32.7 ± 1.81	
	0.250	17.9 ± 0.58	
	0.125	9.71 ± 2.33	
